# Supplementary material for: Identification and functional analysis of non-coding regulatory small RNA FenSr3 in Bacillus amyloliquefaciens LPB-18
Source: PeerJ. 2023 May 15;11:e15236. doi: 10.7717/peerj.15236 (PMC10194069; doi:10.7717/peerj.15236)
Supplement: Supplemental Information 4 [file peerj-11-15236-s004.zip › KO/CK-vs-T1_map/map02030.html]

KEGG PATHWAY: Bacterial chemotaxis - Reference pathway


|  |  |
| --- | --- |
| **Bacterial chemotaxis - Reference pathway** |  |

[
Pathway menu
| Organism menu
| Pathway entry
| Show description
| User data mapping
]

Chemotaxis is the process by which cells sense chemical gradients in their environment and then move towards more favorable conditions. In chemotaxis, events at the receptors control autophosphorylation of the CheA histidine kinase, and the phosphohistidine is the substrate for the response regulator CheY, which catalyzes the transfer of the phosphoryl group to a conserved aspartate. The resulting CheY-P can interact with the switch mechanism in the motor. This interaction causes a change in behavior, such as in direction or speed of rotation of flagella.

|  |  |  |
| --- | --- | --- |
| Reference pathway | 184% 150% 122% 100% 82% 67% 55% | 图片下载 |
